# Supplementary material for: Integrated intra‐ and intercellular signaling knowledge for multicellular omics analysis
Source: Mol Syst Biol. 2021 Mar 22;17(3):e9923. doi: 10.15252/msb.20209923 (PMC7983032; doi:10.15252/msb.20209923)
Supplement: Supplementary file 1 — Appendix [file MSB-17-e9923-s009.pdf]

Appendix for the article *“Integrated intra- and intercellular signaling knowledge for multicellular omics analysis”*

Dénes Türei[1], Alberto Valdeolivas[1], Lejla Gul[4], Nicolàs Palacio-Escat[1,2,3], Michal Klein[6], Olga Ivanova[1], Marton Olbei[4,5], Attila Gábor[1], Fabian Theis[6,7], Dezső Módos[4,5], Tamás Korcsmáros[4,5], Julio Saez-Rodriguez[1,2,*]

[1] Heidelberg University, Faculty of Medicine and Heidelberg University Hospital, Institute of Computational Biomedicine, Bioquant, 69120 Heidelberg, Germany

[2] RWTH Aachen University, Faculty of Medicine, Joint Research Centre for Computational Biomedicine (JRC-COMBINE), 52074 Aachen, Germany

[3] Heidelberg University, Faculty of Biosciences, 69120 Heidelberg, Germany

[4] Earlham Institute, Norwich Research Park, Norwich, NR4 7UZ, UK

[5] Quadram Institute Bioscience, Norwich Research Park, Norwich, NR4 7UQ, UK

[6] Institute of Computational Biology, Helmholtz Zentrum München, Neuherberg 85764, Germany.

[7] Department of Mathematics, Technical University of Munich, Garching 85748, Germany.

* Corresponding author: julio.saez@uni-heidelberg.de

Table of contents

1. Representation of molecular signaling knowledge **2**

2. Joint analysis of intra- and intercellular processes in SARS-CoV-2 infected lung epithelial cancer (Calu3) cells **3**

2.1. Discussion 7

References **8**

Figures **11**

1. Representation of molecular signaling knowledge

In the design of the *network* database of *OmniPath,* we focused on resources that follow the *activity flow* representation where nodes are linked with signed and directed edges representing a certain influence. The alternative *process description* representation describes the underlying processes as biochemical reactions (Le Novère *et al*, 2009). Integrative resources such as *STRING* (Szklarczyk *et al*, 2019), *PathwayCommons (Cerami et al, 2011a)*, *ConsensusPathDB* (Kamburov *et al*, 2013), *PathMe* and *ComPath* (Domingo-Fernández *et al*, 2019) use mostly the major process description resources (e.g. *Reactome* (Jassal *et al*, 2020) and *ACSN* (Kuperstein *et al*, 2015)) and resources with undirected interactions (e.g. *IntAct* (Orchard *et al*, 2014) and *BioGRID* (Oughtred *et al*, 2019)), and only few activity flow resources. For example, *Pathway Commons* works from 22 resources while OmniPath from 66 resources, and they have only seven in common.

However, for many applications, *process description* representation must undergo a conversion to *activity flow* representation (Cerami *et al*, 2011b). This conversion is technically challenging, leads to information loss (Tang *et al*, 2015; Demir *et al*, 2013; Cerami *et al*, 2011c), alters the network topology, and affects downstream applications. On the other extreme, undirected interactions lack information about directionality and stimulatory and inhibitory effects, which are essential for many analytical methods, in particular those that aim to capture causal relationships. Combination of process description resources converted to networks with the large undirected PPI resources *(IntAct, BioGRID)* in other integrative databases, result in a network density much higher compared to *OmniPath.*

In the activity flow representation the interactions are presented as signed and directed edges, regardless of the underlying biochemistry. Due to this abstraction, activity flow has limitations and the stimulatory and inhibitory nature of the interactions can be ambiguous (Appendix Figure S4; (Touré *et al*, 2020b). Despite these limitations, activity flow databases are widely used because their level of abstraction provides a convenient input for multiple analysis techniques (Touré *et al*, 2020a). Importantly, *OmniPath* lays an emphasis on literature curated data and

integrates a number of small resources which otherwise would be difficult to access for data analysis and modeling applications.

Besides interaction data, process description resources include information about complexes, enzyme-substrate relationships, localization and other attributes as an integral part of their data representation. In contrast, *OmniPath* contains these information in separate databases (complexes, enzyme-substrate relationships, annotations), allowing easy access without complicated parsing.

2. Joint analysis of intra- and intercellular processes in SARS-CoV-2 infected lung epithelial cancer (Calu3) cells

In this note, we provide further details and supporting literature for the results obtained in the *SARS-CoV-2* case study and presented in Figure 5a, Figure EV5 and Appendix Figure S2. In addition, we support our choices and discuss the potential limitations of our approach. In this case study, we aim to explore the potential autocrine regulatory effect of ligands overexpressed in *SARS-CoV-2* infection of epithelial lung cancer cells *(Calu3)* on the expression of inflammatory response genes. We used expression data from a recent publication (Blanco-Melo *et al*, 2020).

We first performed a differential expression analysis of *SARS-CoV-2* infected cells versus mock treated controls. This allowed us to carry out a gene set enrichment analysis revealing inflammatory response as one of the most enriched sets (Figure EV5a). We subsequently selected the most relevant genes involved in inflammatory response (Methods). In addition, we selected overexpressed ligands after infection that are likely to be secreted to the extracellular milieu (Methods). We then applied our *OmniPath*-based version of *NicheNet* to rank the overexpressed ligands secreted by infected *Calu3* cells that are most likely to be involved in the regulation of inflammatory response related genes (Methods). Out of a total of 117 overexpressed ligands, we selected the 12 top-ranked ones for subsequent analysis according to

the distribution of correlation values (Figure EV5b) and *nichenetr* guidelines (Browaeys *et al*, 2019). Among them, we found different types of cytokines: interleukins (IL23A and IL1A), tumor necrosis factors (TNF and TNFSF13B) and chemokines (CXCL5, CXCL9 and CXCL10). These proteins are known to be involved in the immune and inflammatory response, hence supporting our *OmniPath*-based approach. Indeed, *NicheNet scores describing the potential influence of the 12 top-ranked ligands on the set of inflammatory genes are significantly higher than on sets of randomly selected genes* (average p-value=3.25e-08 from Fisher's exact tests after 10 cross-validation rounds). These results suggest that our top-ranked ligands regulate to some extent the expression of inflammatory response related genes in *Calu3* cells upon SARS-CoV-2 infection.

NicheNet ranks the ligands based on their potential effect to regulate the whole set of inflammatory response genes (Browaeys *et al*, 2019). To get more detailed functional and mechanistic insights, we next investigated the inter- and intracellular signaling events that can lead to the activation of a particular ligand-target link. First, we explored the *NicheNet* regulatory potential scores between our top-scored ligands and the top inflammatory response target genes according to our *OmniPath*-based prior knowledge network (Figure EV5c). Then, we selected the receptors expressed in *Calu3* cells after infection that can potentially bind our top ranked ligands, i.e. a known interaction is described between them in our ligand-receptor network (methods). The most likely ligand-receptor pairs according to their *NicheNet* prior interaction potential score are displayed in Figure EV5d. We finally inferred the most likely paths connecting some of our top ranked ligands to their inflammatory response target genes (Figure 5a and Methods).

Among the top predicted ligands, we found three C-X-C motif chemokines (CXCL5, CXCL9 and CXCL10). CXCL9 and CXCL10 are well known pro-inflammatory chemokines that participate in the inflammatory response by recruiting immune cells to infected areas (Qin *et al*, 2011). According to our results, these ligands may potentially bind to C-X-C chemokine receptors (CXCR1 and CXCR2) and to the CCR3 receptor (Figure 5a). Then, CXCR1 and CCR3 can both activate MAPK14, a serine/threonine kinase which plays a key role in the signalling responses

to extracellular stimuli such as proinflammatory cytokines or physical stress leading to direct activation of transcription factors (Lee *et al*, 1994). In addition, CXCR1, CXCR2 and CCR3 directly interact with JAK2, activating the STAT transcription factors. In particular, JAK2 mediates the cytokine-driven activation of the FOS transcription factor, which is a key component in the regulation of proinflammatory genes (Lee *et al*, 2004). Consequently, the use of ruxolitinib, a JAK1 and JAK2 inhibitor, has been suggested as a potential way to prevent the harmful effects of the excessive secretion of proinflammatory proteins, the so-called *cytokine storm,* in severe cases of *COVID-19 (Goker Bagca & Biray Avci, 2020)*.

We also identified two interleukins (IL23A and IL1A) among the top predicted ligands. IL23A forms a heterodimeric cytokine by associating with IL12B, the IL-23 interleukin. IL-23 binds to the IL12RB1-IL23R receptor complex and activates the JAK-STAT signaling cascade promoting the production of proinflammatory cytokines. Furthermore, IL-23 induces autoimmune inflammation and its inhibition is the main treatment for *psoriasis,* an autoimmune disease(Fotiadou *et al*, 2018). In our results (Figure 5a), we identified the interaction between IL23A and IL12RB1, and how IL12RB1 directly activates some of the STAT transcription factors (STAT1, STAT3 and STAT4). IL1A is known to play key roles in the regulation of the immune and the inflammatory response. It binds to the interleukin-1 receptor, interaction that was partially recovered in our signaling network (IL1R2, Figure 5a). Then, IL1R2 activates CASP3, whose role in the modulation of cytokine expression and inflammation has been proposed (Martinon & Tschopp, 2004), although is not as straightforward as in the previous discussed examples.

We additionally retrieved some tumor necrosis factors (TNF and TNFSF13B) as top ligands potentially regulating the expression of inflammatory response related genes. The main functions of TNF are the regulation of immune cells and the systemic inflammatory response. Once TNF comes to contact with their potential receptors, the TRADD protein can also bind to the receptor resulting in the potential initiation of three different pathways: activation of the NFKB pathway, activation of the MAPK pathway or induction of death signaling (Wajant *et al*, 2003). Our results capture the interaction between TNF and TRADD to their potential receptor,

TNFRSF21, which in turn activates *RELA* (Figure 5a). The activation of RELA suggests an activation of the NF-kB pathway, known to be active in *SARS-CoV-2* infection (Mahase, 2020). The TNFSF13B gene encodes the B-cell activating factor (BAFF) protein, which can bind to the TNFRSF13C receptor as identified in our results. The interaction between BAFF and its receptors triggers the activation of the classical and non-canonical NF-kB signaling pathway (Gardam & Brink, 2014). In our results, we identified the activation of MAP3K14, which indeed appears to be involved in the activation of the NF-kB complex and its transcriptional activity (Liao *et al*, 2004).

To further characterize the potential medical relevance of these results, we investigated the drugs targeting the genes shown in Figure 5a (Dataset EV14). As expected, we found several compounds used in the treatment of multiple inflammatory diseases such as rheumatoid arthritis, inflammatory bowel disease and multiple sclerosis. Among the most interesting results, we identified minocycline, an antibiotic and anti-inflammatory drug targeting CASP3. CASP3 is a marker of caspase-dependent apoptosis which interestingly shows an increased activity in the presence of the SARS-CoV-2-encoded protein ORF3a *(Ren et al, 2020)*. Minocycline has been very recently proposed to alleviate the effects of SARS-CoV-2 severe infection in the central nervous system (Oliveira *et al*, 2020). In addition, minocycline successfully decreases inflammatory cytokines such as TNF, which is highly expressed in severe COVID19-patients and linked to an increased neurological damage (Sharma *et al*, 2018; Chen *et al*, 2020). It is to note that TNF was identified in our approach as a top ligand regulating the immune response.

Finally, to further support the relevance of the selected ligands versus the remaining overexpressed ligands, we conducted a hypergeometric test on our 12 top-ranked ligands on the list of curated pathways from MsigDB. We set as background the 117 overexpressed ligands after SARS-CoV-2 infection of the Calu-3 cell line. The significantly enriched pathways are shown in Appendix Figure S2. We found some pathways directly related to our list of 12 prioritised ligands (IL23, CXCR3 and IL10 pathways). The NFKB pathway appears as the most enriched process. We described above how this pathway is connected to some of our predicted

ligands and its activation during *SARS-CoV-2* infection. Toll-like receptors are a class of proteins that are well known for their key role in the innate immune system. In addition, we retrieved an enrichment in the dilated cardiomyopathy pathway and in the HSP27 pathway. Interestingly, it has been postulated that the sustained immune activation upon *SARS-CoV-2* infection increases the risk of developing dilated cardiomyopathy in *COVID-19* patients (Komiyama *et al*, 2020). Passive immunization using anti-HSP27 antibodies has been suggested as a potential treatment against the inflammatory complications of *SARS-CoV-2* infection (O'Brien & Sandhu, 2020).

2.1. Discussion

In summary, we studied how the ligands secreted after *SARS-CoV-2* infection could influence the inflammatory response of neighboring cells. We were able to capture known biological processes supported by the literature. These processes and signaling cascades may lead to the exacerbated inflammatory response observed in the most severe *COVID-19* cases. We also explored the drugs known to target the genes involved in these signaling events in order to highlight the potential medical relevance of our results.

The main goal of this section is to emphasize the usefulness of *OmniPath* as a prior knowledge resource to study cell-cell communication through the integration of inter- and intracellular interactions. We consequently decided to investigate a general biological process known to be mediated by cellular communication: the inflammatory response triggered upon viral infection. Several other pathways and biological processes are perturbed by the *SARS-CoV-2* infection, such as fatty acid metabolism (Figure EV5a), but their link to cell communication is not so straightforward. On the other hand, It would have been interesting to narrow down the analysis to a more specific cell-communication mediated process. However, the results could be harder to interpret and lack support from existing literature, hence requiring experimental validations which are out of the scope of this work.

It is to note that all the ligands considered in our *OmniPath*-based *NicheNet* procedure are overexpressed after *SARS-CoV-2* infection. We consequently identified potentially interesting ligands not ranked among the top 12. For instance, Del Valle et al. (Valle *et al*, 2020) found that

high expression of IL6 and TNF are strong predictors of COVID-19 severity and patient survival, independently of other markers or factors. TNF was ranked second in our approach whereas IL6 was not selected among our top hits. Thus, we were able to find some relevant ligands in the context of the *COVID-19* disease, but also missed important ones, as is expected when using an exploratory data analysis method based on our unavoidably incomplete prior knowledge.

References

Blanco-Melo D, Nilsson-Payant BE, Liu W-C, Uhl S, Hoagland D, Møller R, Jordan TX, Oishi K, Panis M, Sachs D, *et al* (2020) Imbalanced Host Response to SARS-CoV-2 Drives Development of COVID-19. *Cell* 181: 1036–1045.e9

Browaeys R, Saelens W & Saeys Y (2019) NicheNet: modeling intercellular communication by linking ligands to target genes. *Nature Methods* doi:10.1038/s41592-019-0667-5

Cerami EG, Gross BE, Demir E, Rodchenkov I, Babur O, Anwar N, Schultz N, Bader GD & Sander C (2011a) Pathway Commons, a web resource for biological pathway data. *Nucleic Acids Res* 39: D685–90

Cerami EG, Gross BE, Demir E, Rodchenkov I, Babur O, Anwar N, Schultz N, Bader GD & Sander C (2011b) Pathway Commons, a web resource for biological pathway data. *Nucleic Acids Res* 39: D685–90

Cerami EG, Gross BE, Demir E, Rodchenkov I, Babur O, Anwar N, Schultz N, Bader GD & Sander C (2011c) Pathway Commons, a web resource for biological pathway data. *Nucleic Acids Res* 39: D685–90

Chen N, Zhou M, Dong X, Qu J, Gong F, Han Y, Qiu Y, Wang J, Liu Y, Wei Y, *et al* (2020) Epidemiological and clinical characteristics of 99 cases of 2019 novel coronavirus pneumonia in Wuhan, China: a descriptive study. *Lancet* 395: 507–513

Demir E, Babur O, Rodchenkov I, Aksoy BA, Fukuda KI, Gross B, Sümer OS, Bader GD & Sander C (2013) Using biological pathway data with paxtools. *PLoS Comput Biol* 9: e1003194

Domingo-Fernández D, Mubeen S, Marín-Llaó J, Hoyt CT & Hofmann-Apitius M (2019) PathMe: merging and exploring mechanistic pathway knowledge. *BMC Bioinformatics* 20: 243

Fotiadou C, Lazaridou E, Sotiriou E & Ioannides D (2018) Targeting IL-23 in psoriasis: current perspectives. *Psoriasis (Auckl)* 8: 1–5

Gardam S & Brink R (2014) Non-Canonical NF-κB Signaling Initiated by BAFF Influences B Cell

Biology at Multiple Junctures. *Front Immunol* 4: 509

Goker Bagca B & Biray Avci C (2020) Overview of the COVID-19 and JAK/STAT Pathway Inhibition: Ruxolitinib Perspective. *Cytokine Growth Factor Rev*

Jassal B, Matthews L, Viteri G, Gong C, Lorente P, Fabregat A, Sidiropoulos K, Cook J, Gillespie M, Haw R, *et al* (2020) The reactome pathway knowledgebase. *Nucleic Acids Res* 48: D498–D503

Kamburov A, Stelzl U, Lehrach H & Herwig R (2013) The ConsensusPathDB interaction database: 2013 update. *Nucleic Acids Res* 41: D793–800

Komiyama M, Hasegawa K & Matsumori A (2020) Dilated Cardiomyopathy Risk in Patients with Coronavirus Disease 2019: How to Identify and Characterise it Early? *Eur Cardiol* 15: e49

Kuperstein I, Bonnet E, Nguyen H-A, Cohen D, Viara E, Grieco L, Fourquet S, Calzone L, Russo C, Kondratova M, *et al* (2015) Atlas of Cancer Signalling Network: a systems biology resource for integrative analysis of cancer data with Google Maps. *Oncogenesis* 4: e160

Lee JC, Laydon JT, McDonnell PC, Gallagher TF, Kumar S, Green D, McNulty D, Blumenthal MJ, Heys JR, Landvatter SW, *et al* (1994) A protein kinase involved in the regulation of inflammatory cytokine biosynthesis. *Nature* 372: 739–746

Lee Y-N, Tuckerman J, Nechushtan H, Schutz G, Razin E & Angel P (2004) c-Fos as a regulator of degranulation and cytokine production in FcepsilonRI-activated mast cells. *J Immunol* 173: 2571–2577

Le Novère N, Hucka M, Mi H, Moodie S, Schreiber F, Sorokin A, Demir E, Wegner K, Aladjem MI, Wimalaratne SM, *et al* (2009) The Systems Biology Graphical Notation. *Nat Biotechnol* 27: 735–741

Liao G, Zhang M, Harhaj EW & Sun S-C (2004) Regulation of the NF-kappaB-inducing kinase by tumor necrosis factor receptor-associated factor 3-induced degradation. *J Biol Chem* 279: 26243–26250

Mahase E (2020) Covid-19: what treatments are being investigated? *BMJ* 368

Martinon F & Tschopp J (2004) Inflammatory Caspases: Linking an Intracellular Innate Immune System to Autoinflammatory Diseases. *Cell* 117: 561–574

O'Brien ER & Sandhu JK (2020) Sex differences in COVID-19 mortality: opportunity to develop HSP27 (HSPB1) immunotherapy to treat hyper-inflammation? *Cell Stress Chaperones* 25: 725–729

Oliveira AC, Richards EM, Karas MM, Pepine CJ & Raizada MK (2020) Would Repurposing Minocycline Alleviate Neurologic Manifestations of COVID-19? *Front Neurosci* 14: 577780

Orchard S, Ammari M, Aranda B, Breuza L, Briganti L, Broackes-Carter F, Campbell NH, Chavali G, Chen C, del-Toro N, *et al* (2014) The MIntAct project--IntAct as a common curation platform for 11 molecular interaction databases. *Nucleic Acids Res* 42: D358–63

Oughtred R, Stark C, Breitkreutz B-J, Rust J, Boucher L, Chang C, Kolas N, O'Donnell L, Leung G, McAdam R, *et al* (2019) The BioGRID interaction database: 2019 update. *Nucleic Acids Res* 47: D529–D541

Qin S, Alcorn JF, Craigo JK, Tjoeng C, Tarwater PM, Kolls JK & Reinhart TA (2011) Epigallocatechin-3-gallate reduces airway inflammation in mice through binding to proinflammatory chemokines and inhibiting inflammatory cell recruitment. *J Immunol* 186: 3693–3700

Ren Y, Shu T, Wu D, Mu J, Wang C, Huang M, Han Y, Zhang X-Y, Zhou W, Qiu Y, *et al* (2020) The ORF3a protein of SARS-CoV-2 induces apoptosis in cells. *Cell Mol Immunol* 17: 881–883

Sharma RK, Oliveira AC, Kim S, Rigatto K, Zubcevic J, Rathinasabapathy A, Kumar A, Lebowitz JJ, Khoshbouei H, Lobaton G, *et al* (2018) Involvement of Neuroinflammation in the Pathogenesis of Monocrotaline-Induced Pulmonary Hypertension. *Hypertension* 71: 1156–1163

Szklarczyk D, Gable AL, Lyon D, Junge A, Wyder S, Huerta-Cepas J, Simonovic M, Doncheva NT, Morris JH, Bork P, *et al* (2019) STRING v11: protein--protein association networks with increased coverage, supporting functional discovery in genome-wide experimental datasets. *Nucleic Acids Res* 47: D607–D613

Tang H, Zhong F, Liu W, He F & Xie H (2015) PathPPI: an integrated dataset of human pathways and protein-protein interactions. *Sci China Life Sci* 58: 579–589

Touré V, Flobak Å, Niarakis A, Vercruysse S & Kuiper M (2020a) The status of causality in biological databases: data resources and data retrieval possibilities to support logical modeling. *Brief Bioinform*

Touré V, Vercruysse S, Acencio ML, Lovering RC, Orchard S, Bradley G, Casals-Casas C, Chaouiya C, Del-Toro N, Flobak Å, *et al* (2020b) The Minimum Information about a Molecular Interaction Causal Statement (MI2CAST). *Bioinformatics*

Valle DMD, Del Valle DM, Kim-Schulze S, Huang H-H, Beckmann ND, Nirenberg S, Wang B, Lavin Y, Swartz TH, Madduri D, *et al* (2020) An inflammatory cytokine signature predicts COVID-19 severity and survival. *Nature Medicine* 26: 1636–1643 doi:10.1038/s41591-020-1051-9

Wajant H, Pfizenmaier K & Scheurich P (2003) Tumor necrosis factor signaling. *Cell Death Differ* 10: 45–65

Figures

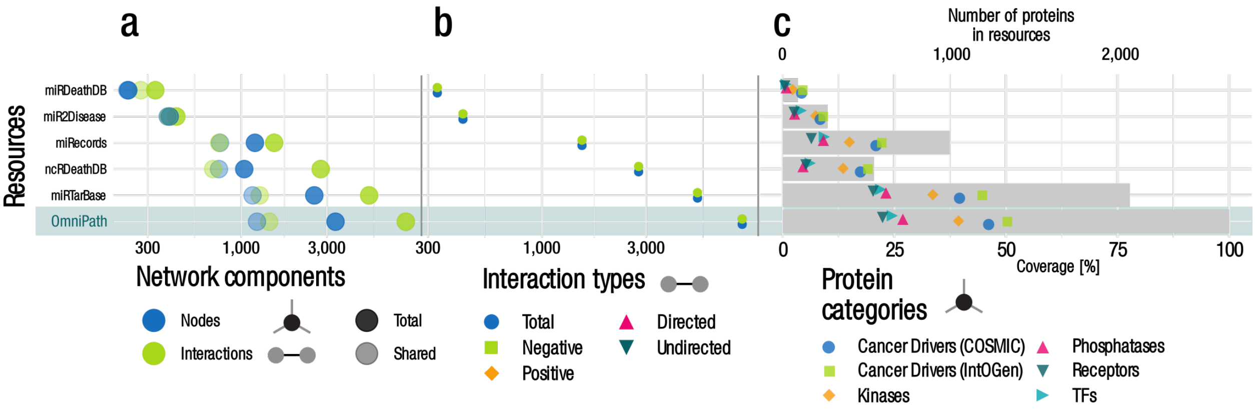

Appendix Figure S1: Quantitative description of the post-transcriptional network by resource. *Panels and notations are the same as on Figure EV1.*

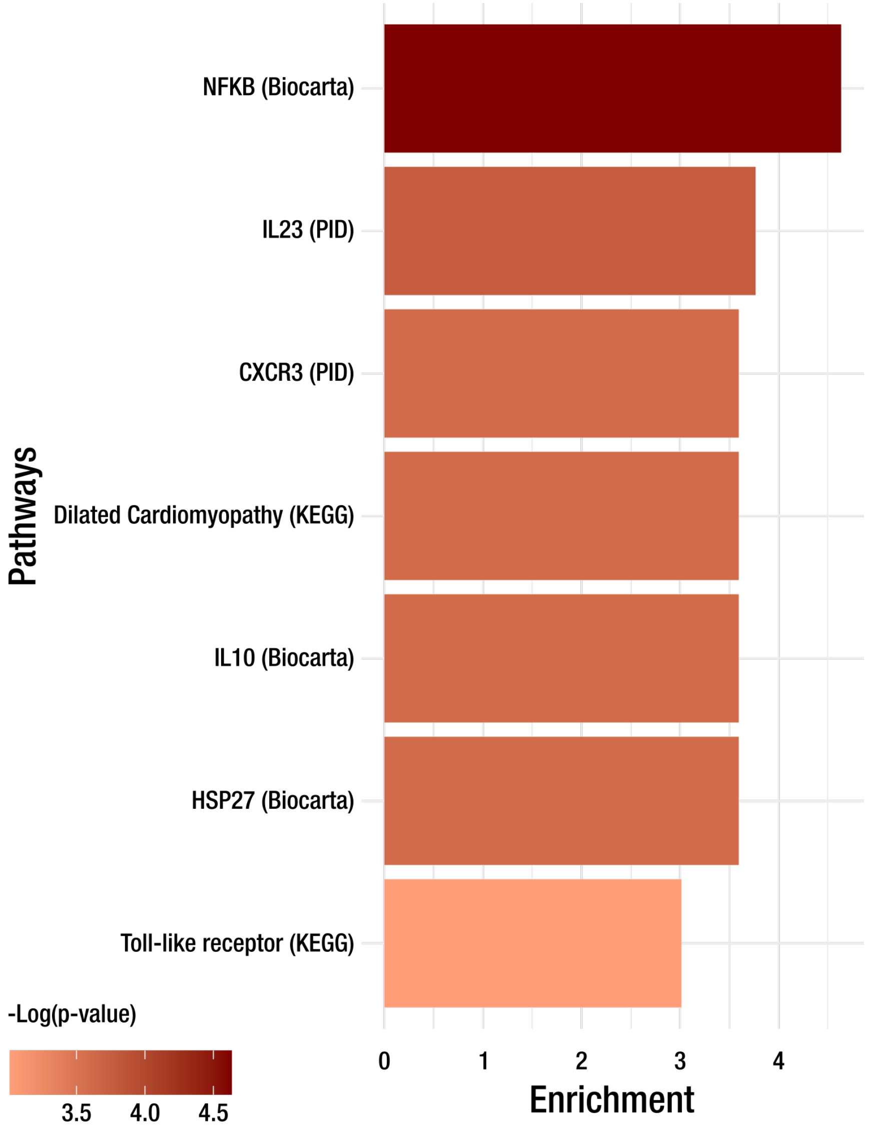

Appendix Figure S2: Pathway enrichment analysis of the 12 ligands with highest NicheNet scores using the 117 ligands overexpressed in SARS-CoV-2 infection as a background. *Hypergeometric test, p < 0.05 shown.*

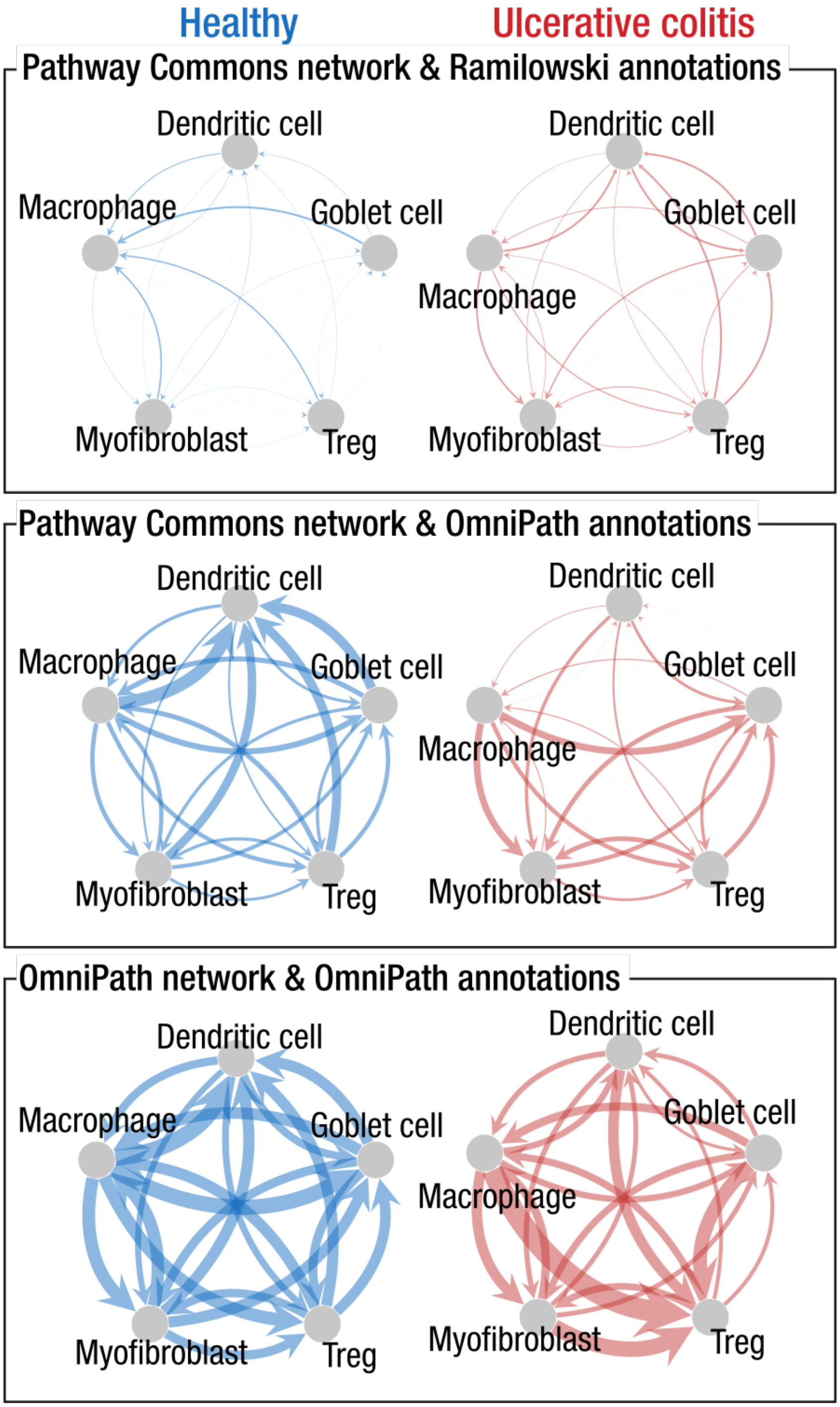

Appendix Figure S3: Cell-cell interactions in healthy condition vs. ulcerative colitis with different database knowledge. *The width of arrows is proportional to the number of connections. Left column: healthy condition; right column: ulcerative colitis. Top row: network connections from Pathway Commons and ligand-receptor annotations from Ramilowski et al; middle row: network from Pathway Commons and ligand-receptor annotations from OmniPath; bottom row: both network and annotations from OmniPath.*

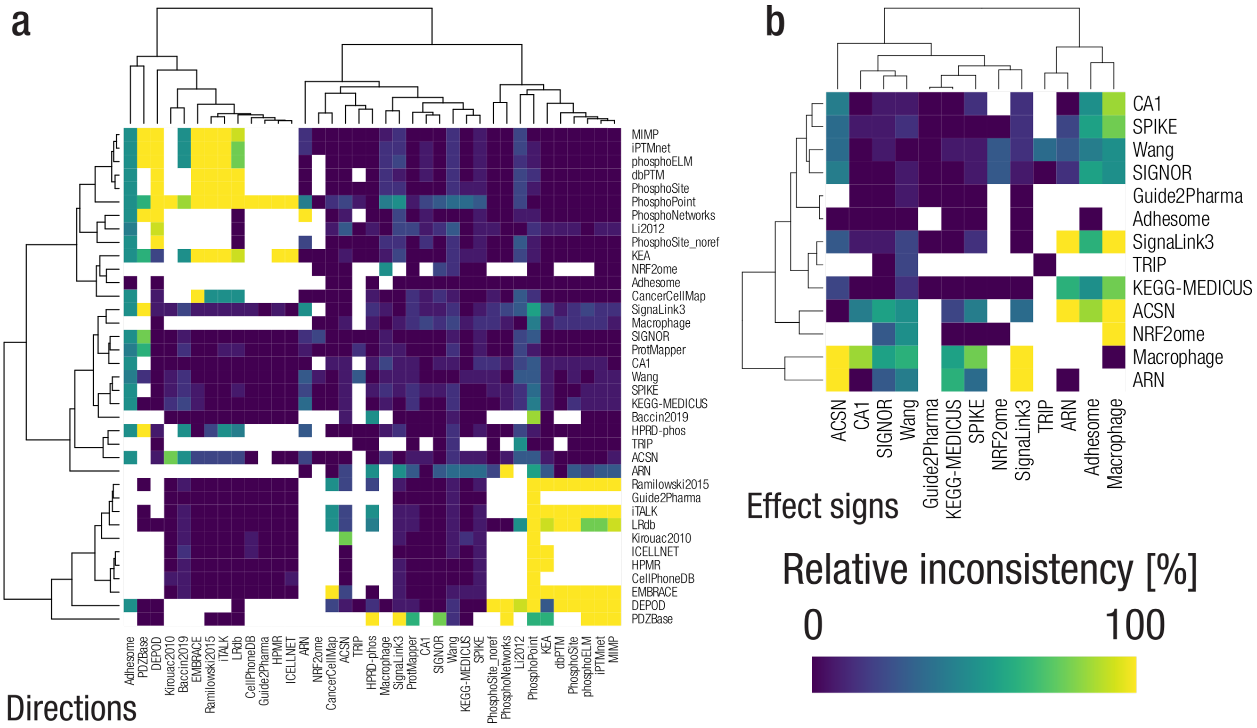

Appendix Figure S4: Pairwise inconsistency of protein-protein interaction resources. *Relative consistency was calculated as the number of inconsistent edges divided by the overlapping edges between two resources. (a) Direction inconsistency: one resource states A affects B while the other states B affects A. (b) Effect sign inconsistency: one resource states A stimulates B while the other states A inhibits B.*

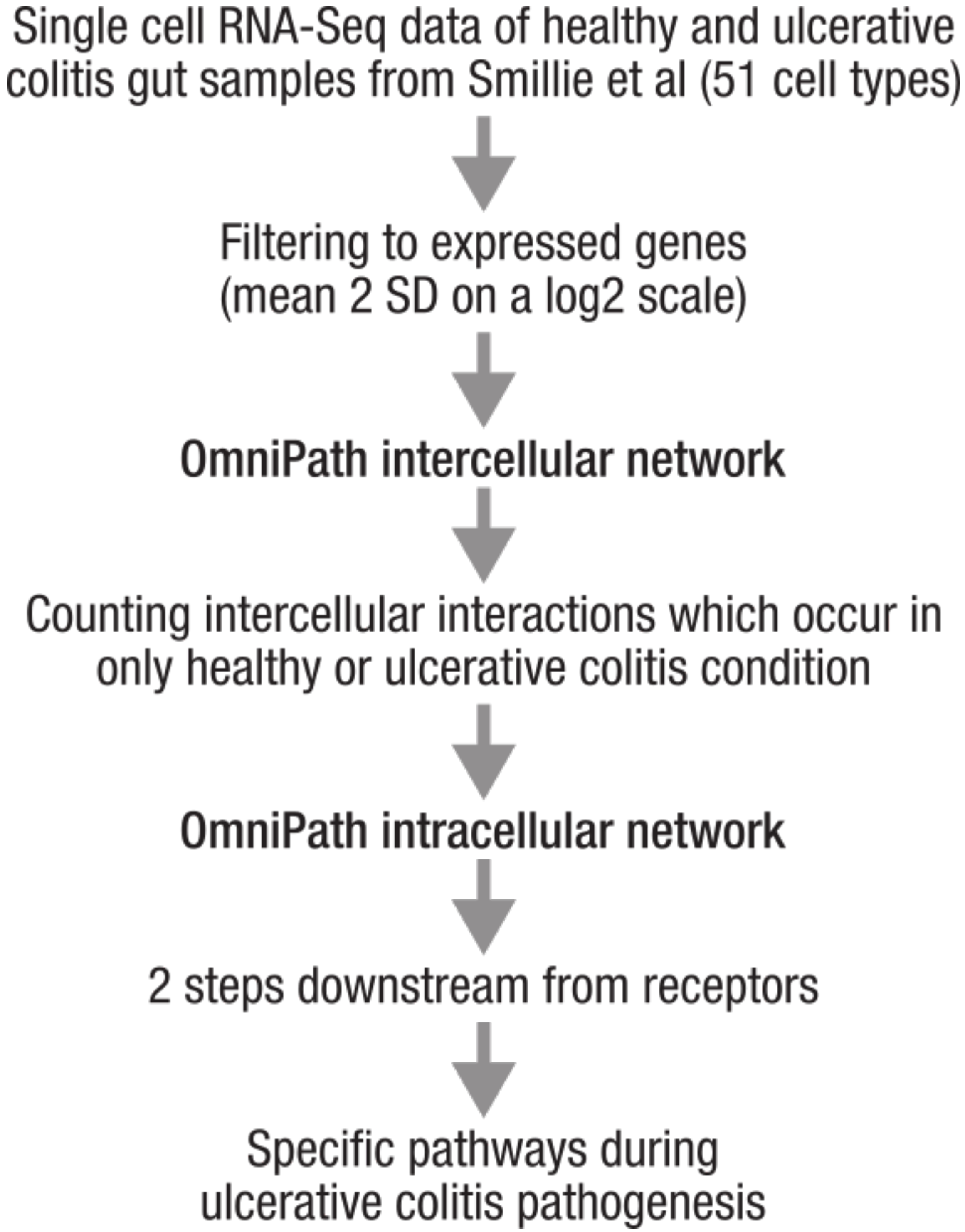

Appendix Figure S5: Workflow of the second case study. *We used scRNA-Seq data from Smillie et al 2019 to study the rewiring of inter- and intracellular pathways in ulcerative colitis. We filtered the interacting partners by their condition specific expression. Then we build intercellular interaction networks between five selected cell types and the intracellular pathways 2 steps downstream of receptors in Treg cells. See more details in the Results.*
