# Supplementary material for: Integrated intra‐ and intercellular signaling knowledge for multicellular omics analysis
Source: Mol Syst Biol. 2021 Mar 22;17(3):e9923. doi: 10.15252/msb.20209923 (PMC7983032; doi:10.15252/msb.20209923)
Supplement: Supplementary file 2 — Expanded View Figures PDF [file MSB-17-e9923-s015.pdf]

Expanded View Figures

Figure EV1. Quantitative description of the PPI network by resource.

A Number of nodes and interactions. The light dots represent the shared nodes and edges (in more than one resource), while the dark ones show their total numbers.
B Causality: number of connections by direction and effect sign.
C Coverage of the networks on various groups of proteins. Dots show the percentage of proteins covered by network resources for the following groups: cancer driver genes from COSMIC and IntOGen, kinases from kinase.com, phosphatases from Phosphatome.net, receptors from the Human Plasma Membrane Receptome (HPMR) and transcription factors from the TF census. Gray bars show the number of proteins in the networks.

© 2021 The Authors

© 2021 The Authors

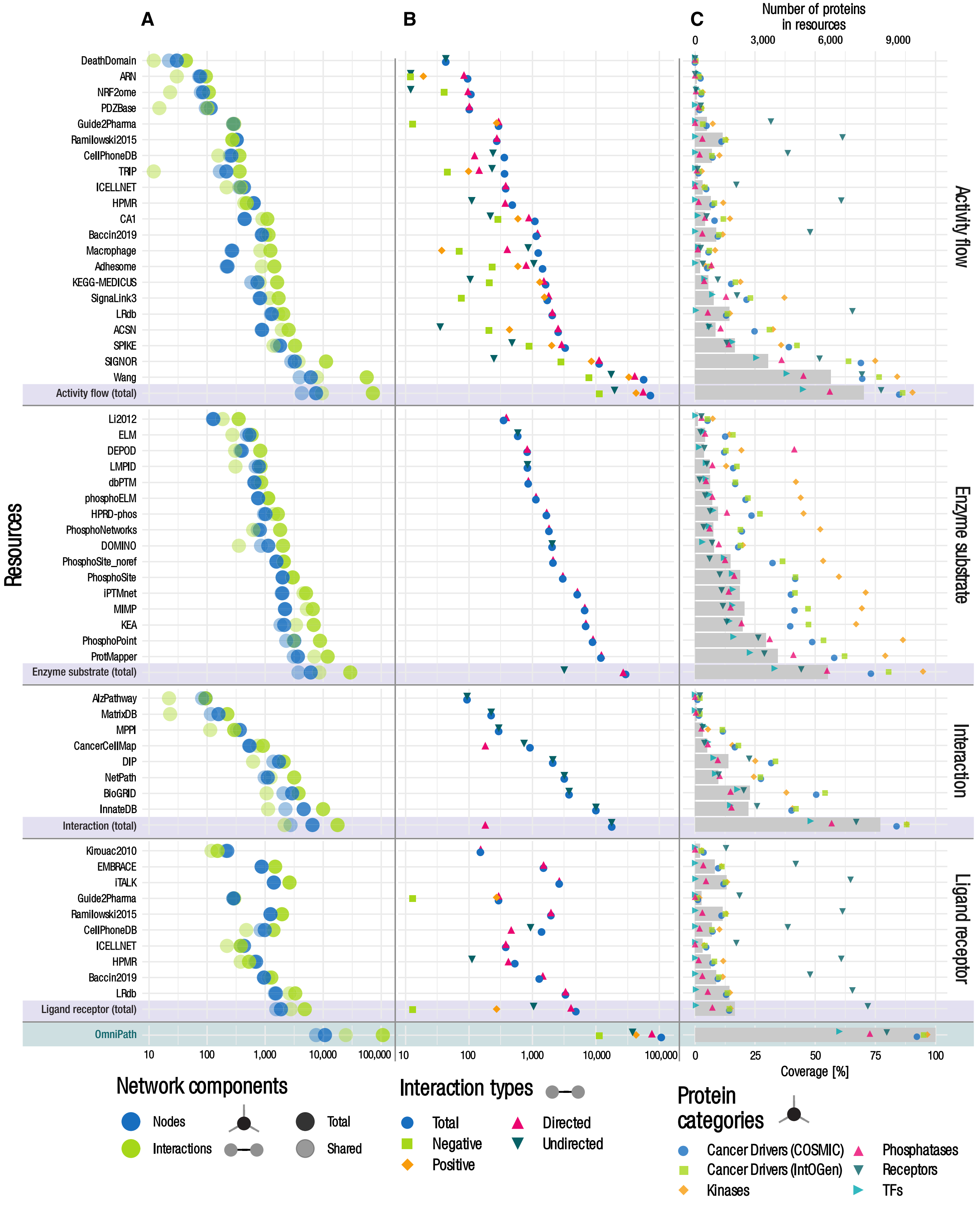

Figure EV1.

© 2021 The Authors

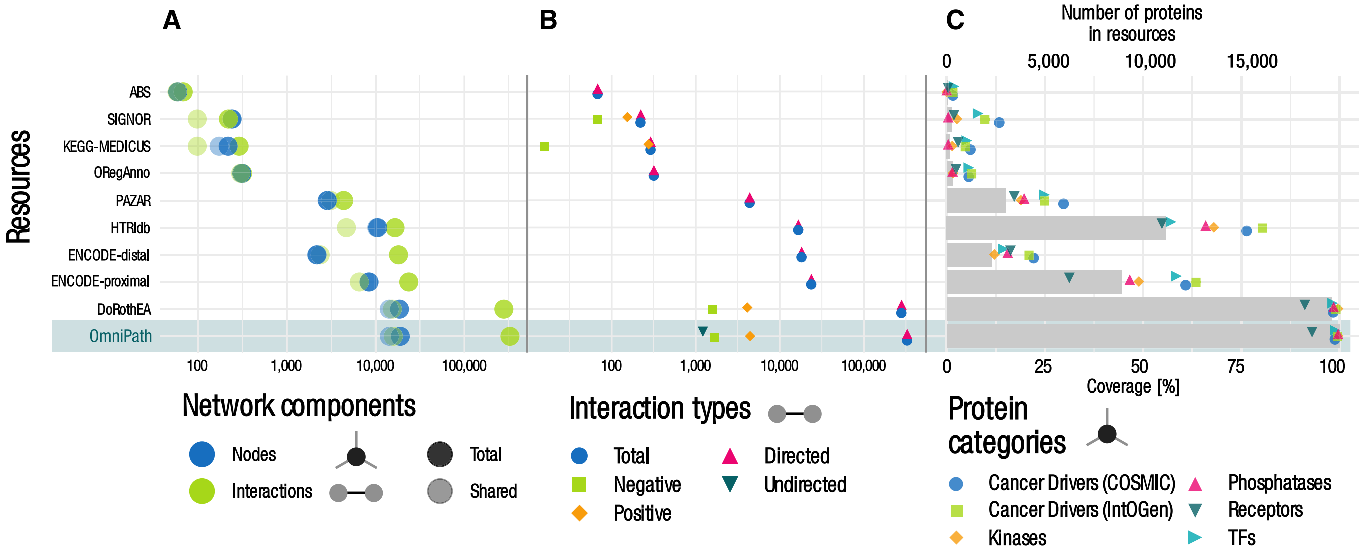

Figure EV2. Quantitative description of the transcriptional network by resource.

A–C Panels and notations are the same as on Fig EV1.

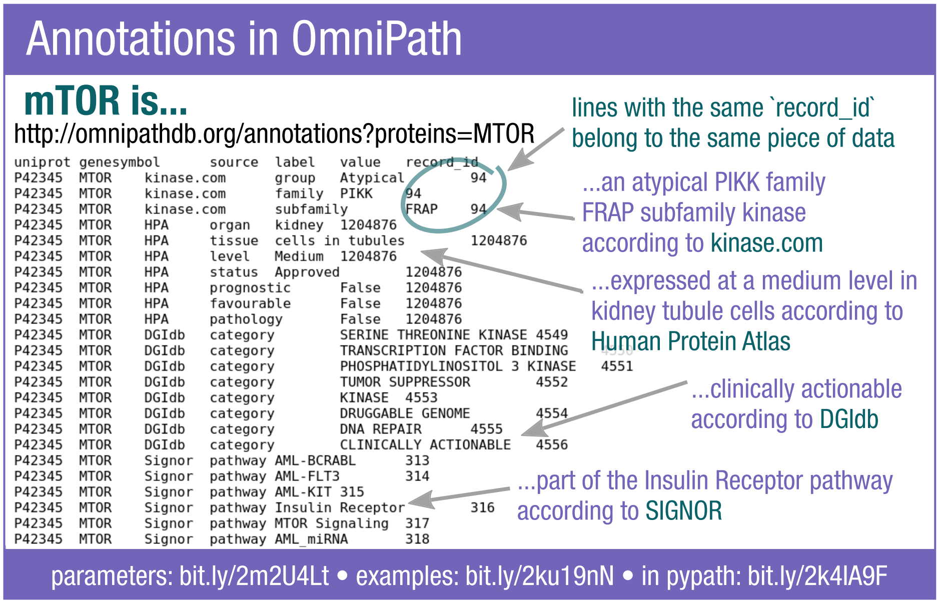

Figure EV3. Example of the annotations query in the OmniPath web service.

For the protein mTOR a large variety of information is available from different databases. The “record_id” binds together the fields of the record from the original resource. Each field has a “label” and a “value”.

© 2021 The Authors

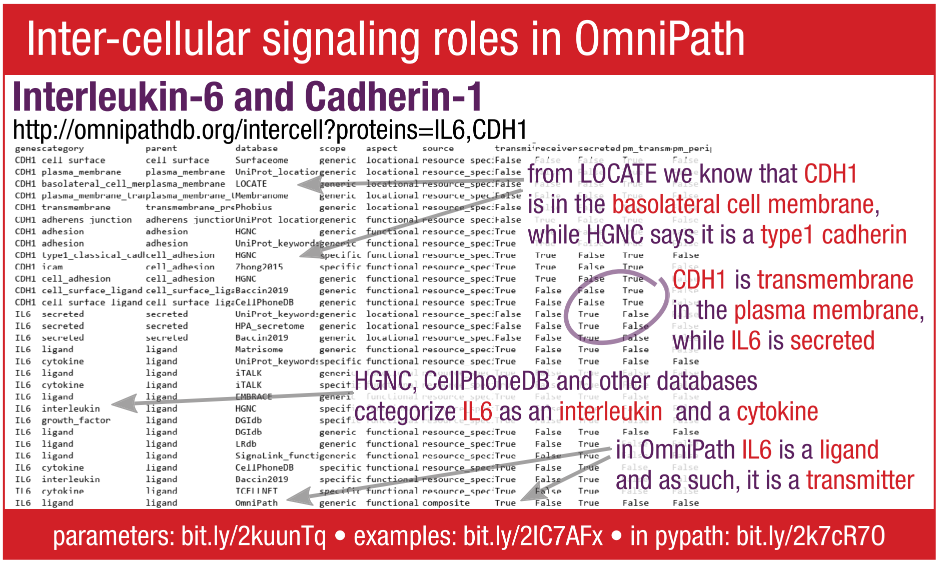

Figure EV4. Example of the intercell query in the OmniPath web service.

Each category has a parent category and a database of origin. The scope of a category is either “generic” (e.g., ligand) or “specific” (e.g., interleukin). The aspect is either “locational” or “functional”. Further attributes show whether the protein is a signal transmitter or a receiver, and whether it is secreted, or a transmembrane or peripheral protein of the plasma membrane.

© 2021 The Authors

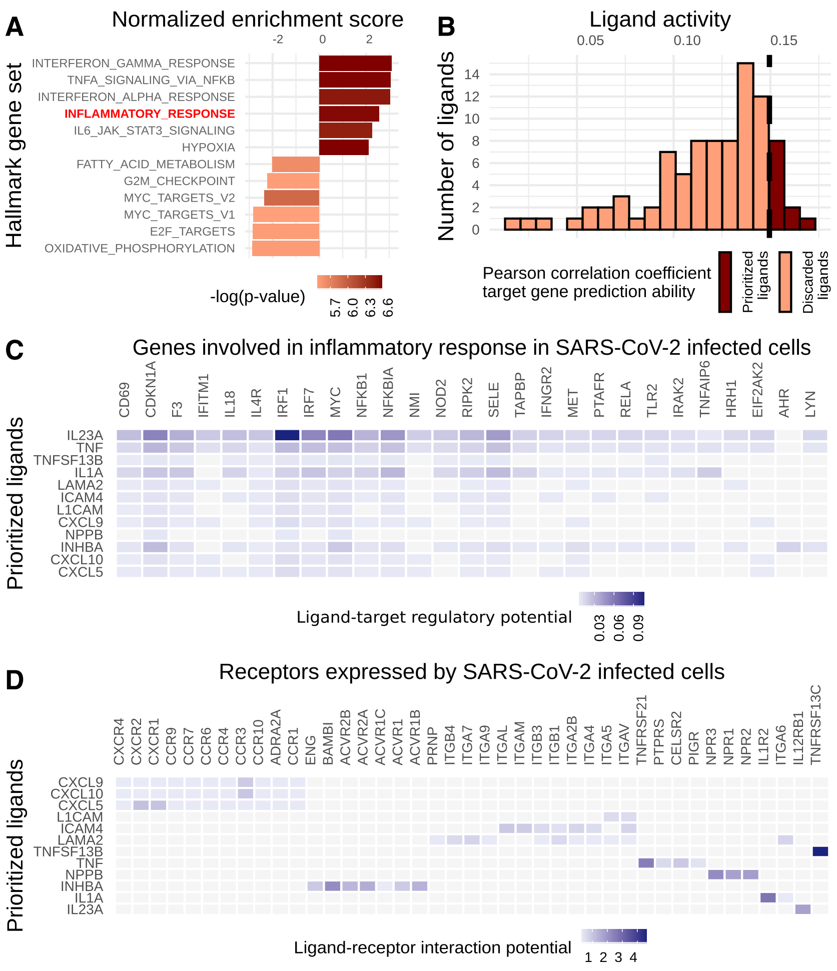

Figure EV5. OmniPath-based NicheNet analysis to predict over-expressed ligands in SARS-CoV-2 infection potentially affecting the expression of inflammatory response related genes in Calu3 cells.

A Most significantly enriched gene sets after SARS-CoV-2 infection on the Calu3 cell line. Inflammatory response is highlighted in red.

B Results of NicheNet's ligand activity analysis: Number of over-expressed ligands after SARS-CoV-2 infection and their potential to predict the inflammatory response gene set based on the Pearson correlation coefficient. The top 12 ranked ligands, out of a total of 117 over-expressed ligands, were selected.

C Regulatory potential of the top ranked ligands and target genes from the inflammatory response program based on NicheNet's prior knowledge model.

D Ligand–receptor interaction potential based on NicheNet's prior knowledge model between the top ranked ligands and the receptors expressed in the Calu3 cell line.

© 2021 The Authors
